# Kitchen Hygiene and Microbial Contamination of Selected Urban Residential Houses in Dhaka City of Bangladesh

**DOI:** 10.1155/ijfo/3380676

**Published:** 2026-04-24

**Authors:** Sharmin Zaman, Md. Ashfaq Aziz, Shiori Katta, Yuna Takimoto, Junichi Sugiyama, Yasushi Kakizawa, Md. Latiful Bari

**Affiliations:** ^1^ Centre for Advanced Research in Sciences, University of Dhaka, Dhaka, Bangladesh, du.ac.bd; ^2^ Research and Technology Center, Lion Corporation, Edogawa-ku, Tokyo, Japan

**Keywords:** food handling, household kitchen, hygiene practices, kitchen utensils, microbial contamination

## Abstract

Food safety in the home kitchen has become a growing public health issue due to inaccurate food preparation processes in consumers’ homes, as well as poor personal and/or environmental hygiene. This study evaluated the prevailing environment, cleanliness, and hygiene habits of selected household kitchens using on‐site visits and swabbing for microbial examinations. Most food contact surfaces, touch points, and kitchen supplies showed high contamination with total aerobic bacterial count (TABC), coliforms, and *Escherichia coli*. Among the food contact surfaces, the highest contamination was recorded in kitchen sink walls (91.4%) with *E. coli*. Approximately 67.0% of food preparation surfaces were contaminated with *E. coli*. Among the frequently touched surfaces, kitchen tap knobs (87.5%) were highly contaminated with *E. coli*. Furthermore, among the frequently used supplies, 91% of sponges/dishcloths were found to be contaminated with *E. coli*. About 81.0% of detergent bars were found to be contaminated with *E. coli*. The majority of homes (93.3%–100.0%) lack access to hot water, soap, and sanitizers in the kitchen, and the presence of pets (cats; 6.7% of households) or pests (100% of homes) in the kitchen was evident, indicating the likelihood of microbial contamination and transmission from the outside environment. To reduce the risk of microbial contamination, people must improve their food handling practices through education.

## 1. Introduction

Understanding food safety behavior at home is necessary for developing effective strategies to mitigate foodborne disease, because foodborne diseases remain a significant public health threat in developing countries like Bangladesh [1, 2]. Although the actual number of foodborne outbreaks caused by home‐produced food is unknown and receives less attention because they affect fewer people (mostly families), many research reports have shown that food consumed at home was the main reason for most foodborne diseases among consumers [3–5]. However, each year, over 30 million individuals suffer from foodborne diseases in Bangladesh [6, 7]. Utilizing the ICDDR,B (International Centre for Diarrhoeal Disease Research, Bangladesh) data, the WHO reported that every day in summer, 501 patients visit a hospital for diarrheal disease treatment attributed to foodborne and waterborne causes [1, 2].

Many studies have found that raw materials are the most common cause of contamination in the kitchen; nevertheless, the areas surrounding the kitchen may also be sources of free‐living bacteria. Enteric pathogens, including *Escherichia coli*, *Salmonella*, *Staphylococcus* spp., *Klebsiella* spp., and *Enterobacter* spp., and opportunistic pathogens like *Pseudomonas* spp. have been isolated [8–10]. Furthermore, commonly used utensils such as cutting boards, knives, dishcloths/sponges, sink surfaces, food preparation surfaces, the detergent used for washing, and the kitchen supply water can be potential sites of microbial reservoirs, and bacteria have been observed to persist in these areas of the kitchen [10–12]. The kitchen food preparation surfaces and cutting boards were found to be contaminated with *E. coli* (> 10^3^ CFU/swab) [13]. Although the majority of purchased goods are regarded as safe, consumer food handling behavior at home is seen as the ultimate line of defense against foodborne infections [4, 14]. Pathogens can enter the home through naturally contaminated raw food, environmental transfer brought by consumers, animals, or insects, or human transfer via food handling and the respiratory route [15]. Family members do not consider themselves to be a source of contamination or to be vulnerable to the dangers of foodborne diseases, and many cases are not reported to health authorities. Some foodborne diseases have few symptoms, and patients may not seek medical attention, contributing to the underreporting of epidemiological data, resulting in a lack of public education direction and training activities [16, 17].

Despite concerns about the occurrence of foodborne infections at home, research on food safety concerns in home kitchens is still scarce [18, 19]. On the other hand, Bangladeshi consumers are particularly vulnerable to microbiological food hazards due to their dense population and low‐ to middle‐income status. Although good personal hygiene and sanitary handling practices help reduce these hazards, Bangladeshi consumers have inadequate food safety knowledge among domestic food handlers [20]. Therefore, consumers’ food safety knowledge, attitudes, and behaviors must be addressed in order to control foodborne infections in home‐prepared food.

A number of approaches have been developed to evaluate food safety behavior at home, such as self‐reported information on consumers’ attitudes, knowledge, and behaviors [21, 22], direct observation of food safety behaviors at home [23], and food handling skills [24, 25], but all these methods have several disadvantages because every type of observation has the potential to influence a subject’s behavior, and the research design must ensure that the participant exhibits his or her “usual” behavior rather than a perceived “correct” behavior [23]. Thus, a visual inspection and swab collection can objectively and methodically identify potential food safety issues in consumer households. The purpose of this study is to examine food safety concerns and food hygiene status in selected household kitchens in Dhaka in order to develop an effective food safety education plan that includes measuring consumer knowledge, detecting misconceptions, and motivating behavior change.

## 2. Methods and Materials

The Centre for Advanced Research in Sciences (CARS) of the University of Dhaka review board approved all of the materials and techniques used in this study. Twelve police stations (Sher‐e‐Bangla Nagar, Ramna, Mirpur, Shahbagh, Kafrul, Lalbagh, Jatrabari, Motijheel, Shyampur, Uttara [East and West], and West Shewrapara) of Dhaka City were randomly selected for visual assessment and microbiological analysis. All participants provided informed consent prior to sample collection and visual assessment.

### 2.1. Sample Size

A visual food safety risk assessment was conducted, as well as the collection of swab or liquid samples for microbiological examinations from chosen participant residences. Microbiology graduate students, one male and one female, were divided into two groups to visit five residences per week and collect samples. Similarly, two groups were formed for laboratory analysis. The samples were collected and analyzed between March and June 2024, with a target population of 100 families drawn from the researchers’ relatives and acquaintances of varying addresses, ages, and household incomes.

### 2.2. Selection of Home Kitchens and Visits

As the kitchen of a home is a highly sensitive place in Bangladeshi culture, relatives of researchers, colleagues, friends and their families, students’ relatives, and acquaintances’ homes were predominantly chosen for this study. The interested households were given a brief explanation of the study’s goal, the collection of swab/liquid samples from 14 kitchen sites, and a visual food safety hazard assessment. Households willing to allow student volunteers to enter their kitchens were chosen. The selected homeowner was requested to make an appointment for their kitchen visit, and phone calls were made to remind them of their appointment several days in advance. To ensure consistency, appointments for kitchen visits were restricted to weekdays between 8 a.m. and 8 p.m. Households were discouraged from preparing meals or cleaning their houses in ways other than their usual routines. Informal training sessions were held at regular intervals throughout the trial to discuss the instructions and protocol for utilizing the visual food safety hazard assessment questionnaires. All data collection was undertaken by two groups of microbiology students (each group comprised a male and a female). They separately documented observations in each house using the assessment questionnaires. The students did not discuss their observations, instead referring to the assessment questionnaire instructions to determine how to evaluate the current circumstance. The visual assessment and swab collection took about 40 min to complete.

### 2.3. Visual Assessment and Rating

The modified visual food safety hazard assessment questionnaires were used in this investigation as reported by Byrd‐Bredbenner et al. [11]. There are 13 key questions on food safety risks in individual home kitchens, with “yes” and “no” responses. When both raters agreed, an individual observation was taken into account. This method was used to obtain a conservative estimate of the actual observations. Furthermore, 14 major kitchen utensils were observed, and the microbiological results (total aerobic bacterial count [TABC], total coliform count [TCC], and *E. coli*) of swabs from each utensil were taken into account. The presence of more than 2.0 log CFU/cm^2^ (colony‐forming units per square centimeter) of TABC, 1.0 log CFU/cm^2^ of TCC, and 1.0 log CFU/cm^2^ of *E. coli* on utensil surfaces was considered a food safety concern [26, 27]. These threshold values were adopted from previously published studies that evaluated domestic kitchen hygiene and were used here as practical cutoffs to flag potential food safety concerns. All data were computed and expressed as percentages.

### 2.4. Swab Sample Collection

**Figure 1:**
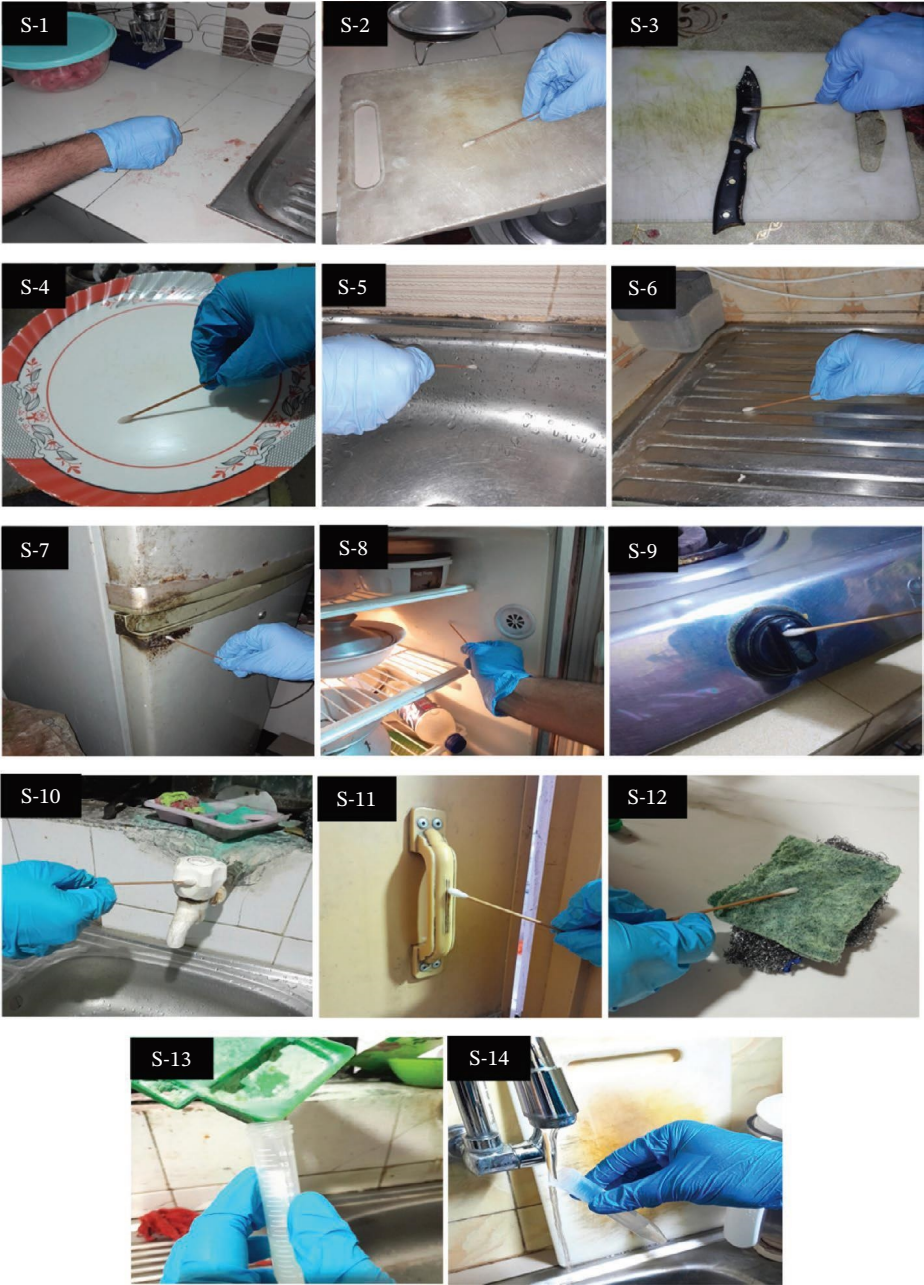
Sampling points for the selected utensils and items in a residential kitchen.

A group discussion was held with three food researchers to analyze contamination risks, five food business operators to share insights into hygiene standards, three consumer association groups, and four working housewives to provide practical perspectives on daily practices and also to determine the sampling points for determining the microbiological status of kitchen hygiene, yielding 14 sampling points, as shown in Table 1 and Figure 1. In this study, 100 private homes in Dhaka City were investigated, and swabs were collected from 12 predetermined places and liquid (1 mL) from two defined points in each household. Every day, two home kitchen samples were obtained based on the householders’ preferences, and swabs from each site were taken following the routine daily cleaning of the home, using one cotton swab soaked with buffered peptone water (BPW, Oxoid). Swab samples were taken in 10 × 10‐cm areas wherever possible; otherwise, the surface area was measured, and the results were expressed in CFU/cm^2^. CFU/cm^2^ is a unit of measurement used in microbiology to quantify the concentration of viable microorganisms present on a surface. All the collected samples were transported to the laboratory in a cool box within 2 h after collection, refrigerated, and processed within 24 h.

**Table 1:** Samples collected from 100 selected households in Dhaka City.

Sample number	Sample location		Sample type
K_S1:	Food preparation surfaces	Common food contact surfaces	Swab sample
K_S2:	Cutting boards		Swab sample
K_S3:	Cutting knives		Swab sample
K_S4:	Serving plates		Swab sample
K_S5:	Kitchen sink walls		Swab sample
K_S6:	Kitchen sink surfaces		Swab sample
K_S7:	Refrigerator door handles	Frequently touched surfaces	Swab sample
K_S8:	Refrigerator internal walls		Swab sample
K_S9:	Cooking stove knobs		Swab sample
K_S10:	Kitchen tap knobs		Swab sample
K_S11:	Toilet doorknobs/handles		Swab sample
K_S12:	Washing steel/dish sponge/cloth	Frequently used supplies	Swab sample
K_S13:	Detergent bar containing pot water		Liquid sample
K_S14:	Kitchen supply water		Liquid sample
	**T** **o** **t** **a** **l** **n** **u** **m** **b** **e** **r** **of** **s** **a** **m** **p** **l** **e** **s** = 14 **f** **r** **o** **m** **e** **a** **c** **h** **h** **o** **u** **s** **e** **h** **o** **l** **d**		

### 2.5. Microbiological Analyses

All swab samples containing BPW tubes were serially diluted, and diluted and nondiluted samples were vortexed, and 100‐*μ*L aliquots were surface plated onto Tryptic Soya Agar (Becton Dickinson and Company, New Jersey, United States) for the determination of TABC, Chromocult Agar (Merck, Darmstadt, Germany) for TCC, and Cefexime Tellurite Sorbitol MacConkey Agar (CT‐SMAC; Oxoid, New Hampshire, United Kingdom) for *E. coli*. All agar plates were incubated at 37°C for 24–48 h before the presumptive colonies were counted. Presumptively identified *E. coli* colonies on CT‐SMAC were enumerated, and a subset was biochemically confirmed using API 20E test kits.

### 2.6. Statistical Analysis

Reported plate count data represent the mean values obtained from individual trials, with each of these values being obtained from duplicate samples. Significant differences in plate count data were established by the least significant difference at the 5% level of significance. Responses to visual assessment questions were numerically entered into an Excel spreadsheet and were calculated and expressed in percentages. Pearson’s correlation analysis was used to determine if there were statistically significant differences in microbial contamination across different surface categories. Analyses were exploratory and limited by sample size and observational design. Furthermore, correlation analyses with and without *E. coli* in household objects were conducted to explore the possible patterns of contamination between household items.

## 3. Results

The majority of the assessed household kitchens lacked adequate lighting. A visual assessment using a set of questionnaires and swab sampling of the kitchen utensils and surfaces was conducted to document the presence of potential food safety hazards in consumer homes. A total of 100 urban household kitchens were selected, and swab samples were collected and analyzed for their TABC, TCC, and the presence of *E. coli* to understand the cleanliness and sanitary practices in these household kitchens.

### 3.1. Visual Food Safety Risk Assessment of Urban Home Kitchens

Several necessary items for effective cleaning, such as soap, towels, sanitizers, and the presence of animals or pests, as well as the cleanliness of food preparation surfaces, cutting boards and knives, kitchen sink walls and surfaces, serving plate surfaces, sponges/dishcloths, and refrigerator exteriors and interiors, were evaluated. It was observed that 91.4% of household kitchen sinks were not thoroughly cleaned, while 67.0% of food preparation surfaces were found unclean. The majority (93.3%–100.0%) of homes do not have soap or hot water in the kitchen, despite the fact that these items are required to properly wash hands, kitchen utensils, and food preparation surfaces. A larger proportion of homes lacked cleaning supplies (100%) or multiple‐use towels in the kitchen (96.7%), and none of the home kitchens contained solely paper towels. These elements are associated with the efficacy of cleaning and hand washing, and they may prevent the survival and/or spread of microbial contamination.

The presence of animals in the kitchen, as well as evidence of pest infestation, is indirectly related to the cleanliness and sanitation of surfaces and animals. Pet food bowls and accessories are present in 6.7% of households. Pest infestation was defined as the presence of any of the following: living or dead insects or rodents (excluding pets), pest‐created material (droppings, nests, and webs), and pest‐eliminating or repelling products (mouse traps and insecticide treatments). Pest infestation was detected in 100% of houses, with the presence of insects (62%), insect repellents, poison, or traps (26%), and mouse poison/traps (12%). Ants were the most common pests found in consumer houses, but cockroaches, flies, mosquitoes, and spiders were also seen. The presence of pets or pests in food preparation facilities may raise the risk of contamination and the transfer of diseases from the outside environment into the home.

### 3.2. Microbial Contamination in Commonly Used Food Contact Surface Items in the Kitchen

**Figure 2:**
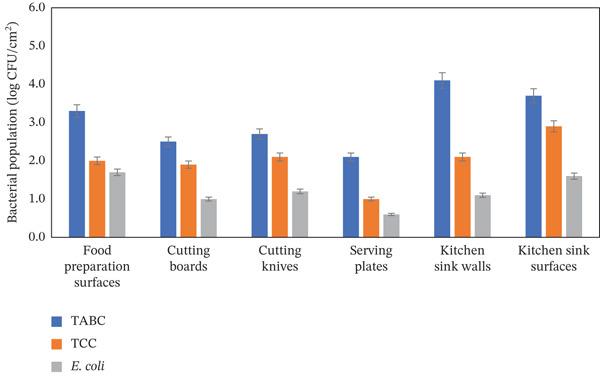
Average microbial population in commonly used food contact surface items in the household kitchen.

The food preparation surfaces, cutting boards, cutting knives, kitchen sink surfaces, kitchen sink walls, and serving plate surfaces were identified as commonly used food contact surface items in the kitchen. The cleanliness and sanitization of these food contact surfaces are important to prepare and serve safe food for families. However, all these food contact surfaces were found to be grossly contaminated with multiple bacteria. Among the food contact surfaces, about 67.0% of food preparation surfaces were found to be contaminated with *E. coli* (1.7 ± 0.1 log CFU/cm^2^), TCC (2.0 ± 0.1 log CFU/cm^2^), and TABC (3.3 ± 0.2 log CFU/cm^2^). The kitchen sink walls showed the highest contamination (91.4%) with*E. coli* (1.1 ± 0.1 log CFU/cm^2^), TCC (2.1 ± 0.1 log CFU/cm^2^), and TABC (4.1 ± 0.2 log CFU/cm^2^). About 87.0% of the kitchen sink surfaces were found to be contaminated with *E. coli* (1.6 ± 0.1 log CFU/cm^2^), TCC (2.9 ± 0.1 log CFU/cm^2^), and TABC (3.7 ± 0.2 log CFU/cm^2^) (Table 2 and Figure 2). On the other hand, about 40.0% of cutting boards and 48.0% of cutting knives were found to be contaminated with *E. coli* ranging from 1.0 to 1.2 ± 0.1 log CFU/cm^2^, TCC ranging from 1.9 to 2.1 ± 0.1 log CFU/cm^2^, and TABC ranging from 2.5 to 2.7 ± 0.1 log CFU/cm^2^, while the lowest contamination was found in serving plates (34.0%) with *E. coli* (0.6 ± 0.0 log CFU/cm^2^), TCC (1.0 ± 0.1 log CFU/cm^2^), and TABC (2.1 ± 0.1 log CFU/cm^2^) (Table 2 and Figure 2). This finding demonstrates the need for appropriate cleaning and sanitization in removing harmful bacteria from food contact surfaces.

**Table 2:** Visual inspection of food safety hazard conditions and *E. coli* contamination in commonly used kitchen utensils or surfaces observed in consumer homes.

Food safety risk	% of homes with food safety concerns (based on visual inspections)	% of *E. coli* & TCC (> 1.0 log CFU/cm^2^) detection rate^*^ (based on swab results)
No hot water in the home	100.0	—
Sanitizing and/or disinfecting cleaners not available	97.7	—
Lack of towels (paper or cloth) in the kitchen	96.7	—
Lack of paper towels in the kitchen	100.0	—
Lack of cutting boards in the kitchen	16.7	—
No soap available at the kitchen sink	93.3	—
Dishes, kitchenware, and/or utensils present in the kitchen sink	30.0	—
Lack of paper/hand towels in the bathroom	0	—
No soap available at the bathroom sink	0	—
Animal(s) present in the food preparation/consumption area	6.7	—
Evidence of pest infestation	100.0	—
Cutting boards are worn with deep grooves/cracks on the surface	55.0	—
Cutting boards (28/70)	—	40.0
Food preparation surfaces (67/100)	—	67.0
Cutting knives (48/100)	—	48.0
Serving plates (34/100)	—	34.0
Kitchen sink walls (64/70)	—	91.4
Kitchen sink surfaces (87/100)	—	87.0
Kitchen tap knobs (35/40)	—	87.5
Toilet doorknobs/handles (21/40)	—	52.5
Washing steel/dish sponge/cloth (91/100)	—	91.0
Detergent bar containing pot water (81/100)	—	81.0
Kitchen supply water (4/39)	—	10.3
Refrigerator door handles (12/40)	—	30.0
Refrigerator internal walls (9/40)	—	22.5
Cooking stove knobs (16/40)	—	40.0

^*^The presence of *E. coli* and TCC > 1.0 log CFU/cm^2^.

^**^The percentages were calculated based on the number of households that possessed the specific item or sampling point, not always the full 100 households. Therefore, the parameter differs across items such as refrigerator‐related surfaces, cutting boards, or other sample‐specific kitchen materials.

### 3.3. Microbial Contamination in Frequently Touched Items

**Figure 3:**
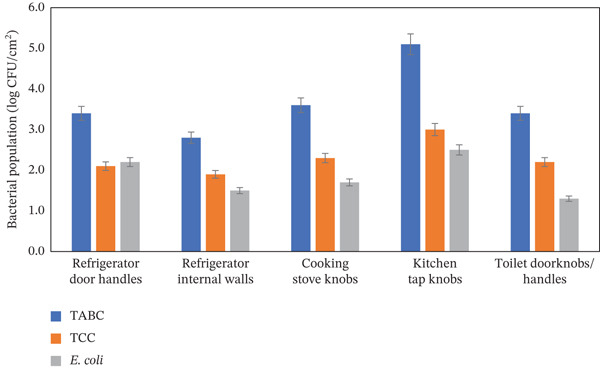
Average microbial contamination in frequently touched surfaces of urban household items.

The frequently touched surfaces in an urban household associated with contamination include refrigerator door handles, refrigerator interior surfaces, kitchen stove knobs, kitchen tap knobs, and toilet doorknobs, which are typically ignored for cleaning or sanitizing and thus have the potential to cross‐contaminate food products. Among the frequently touched surfaces, 87.5% of the kitchen tap knobs were found to be contaminated with *E. coli* (2.5 ± 0.3 log CFU/cm^2^), higher TCC (3.0 ± 0.1 log CFU/cm^2^), and TABC (5.1 ± 0.2 log CFU/cm^2^). Approximately 40.0% of cooking stove knobs were found to be contaminated with *E. coli* (1.7 ± 0.2 log CFU/cm^2^), TCC (2.3 ± 0.3 log CFU/cm^2^), and TABC (3.6 ± 0.1 log CFU/cm^2^) (Table 2 and Figure 3). This finding implies that the kitchen tap knobs and cooking stove knobs that are not cleaned or sanitized have the potential to cross‐contaminate home‐prepared food. In addition, about 30.0% of home refrigerator door handles and 22.5% of refrigerator internal surfaces were found to be contaminated with *E. coli* ranging from 1.5 to 2.2 ± 0.4 log CFU/cm^2^, TCC ranging from 1.9 to 2.1 ± 0.4 log CFU/cm^2^, and TABC ranging from 2.8 to 3.4 ± 0.1 log CFU/cm^2^. This finding suggests that the refrigerator door handles and internal walls should be cleaned and sanitized on a regular basis to improve food safety at the household level. Furthermore, about 52.5% of the toilet doorknobs were found to be contaminated with *E. coli* (1.3 ± 0.1 log CFU/cm^2^), TCC (2.2 ± 0.1 log CFU/cm^2^), and TABC (3.4 ± 0.1 log CFU/cm^2^) (Table 2 and Figure 3), showing inadequate personal hygiene practices in the home. All of these frequently touched household items indicate that a potential food safety risk persists in kitchen hygiene practices in Bangladeshi urban families.

### 3.4. Microbial Contamination in Frequently Used Supplies

**Figure 4:**
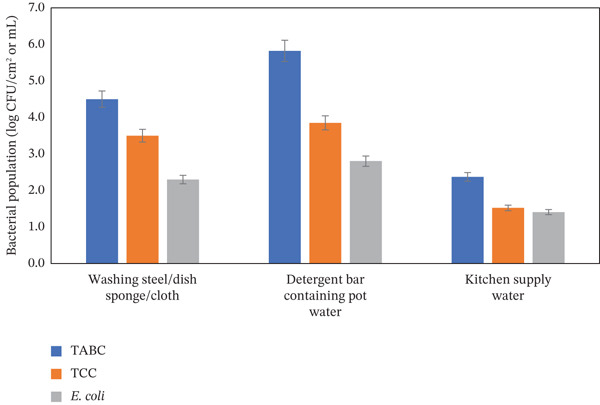
Average microbial population in frequently used supplies in the household kitchen.

The frequently used supplies in the kitchen were identified as supply water, sponges/dishcloths, and detergent bars, which are used to clean kitchen utensils before reuse. About 10.3% of sampled household kitchen supply water was found to be contaminated with *E. coli* (1.4 ± 0.6 log CFU/mL), TCC (1.5 ± 0.6 log CFU/mL), and TABC (2.4 ± 0.8 log CFU/mL). This finding demonstrates that the presence of *E. coli* contamination in kitchen supply water can raise the likelihood of additional contamination (Table 2 and Figure 4). On the other hand, 91.0% of dishwashing sponges/cloths were found to be contaminated with *E. coli* (2.3 ± 0.1 log CFU/cm^2^), TCC (3.5 ± 0.1 log CFU/cm^2^), and TABC (4.5 ± 0.1 log CFU/cm^2^) (Table 2 and Figure 4). This finding demonstrated that using contaminated dishwashing sponges/cloths contributes to further contamination rather than eliminating bacteria. Furthermore, 81.0% of detergent bars were found to be contaminated with *E. coli* (2.8 ± 1.4 log CFU/mL), TCC (3.8 ± 1.4 log CFU/mL), and TABC (5.8 ± 0.9 log CFU/mL), showing that the detergents used for cleaning included a higher number of microorganisms (Table 2 and Figure 4). Although the pH of the detergent bars was highly alkaline (pH 10.5), the way they are used in the family kitchen may lower the pH, allowing bacteria to survive or grow; this mechanism was not directly tested in the present study.

These findings revealed that the domestic kitchen in sampled households was highly contaminated with fecal indicator bacteria and higher coliform and TABC levels, implying that cleaning kitchen utensils with bacteria‐containing detergents and washing them with contaminated supply water increase the risk of contamination rather than reducing bacteria on the utensil surfaces. Therefore, detergents containing sanitizing agents are essential to completely inactivate microorganisms on utensil surfaces, thereby ensuring cleaning and sanitization of the kitchen utensils to prepare and serve safer food for families.

### 3.5. Correlation Analysis (Surface Categories vs. Microbial Contamination and Transmission of *E. coli*)

A correlation analysis was conducted to examine relationships between microbial contamination measures (TABC, TCC, and *E. coli*) across 14 kitchen sampling points. Strong positive correlations were found between all pairs: TABC and TCC (*r* = 0.94, *p* < 0.001), TABC and *E. coli* (*r* = 0.91, *p* < 0.001), and TCC and *E. coli* (*r* = 0.97, *p* < 0.001) (Table 3). These results indicate that contamination levels across different microbial groups are highly consistent in kitchen environments. The data showed strong correlations between all microbial measures, indicating consistent contamination patterns across different kitchen surfaces. This suggests that hygiene interventions affecting one microbial group will likely affect all three similarly.

**Table 3:** Correlation by surface categories.

Category	TABC–TCC *r*	TABC–*E. coli* *r*	TCC–*E. coli* *r*	*n*
Surfaces and items	0.921	0.857	0.943	6
Frequently touched	0.976	0.967	0.998	5
Cleaning and water	0.999	0.995	0.999	3
Overall Pearson’s *r*	0.942	0.912	0.970	14
*p* value	<0.001	<0.001	<0.001	

**Figure 5:**
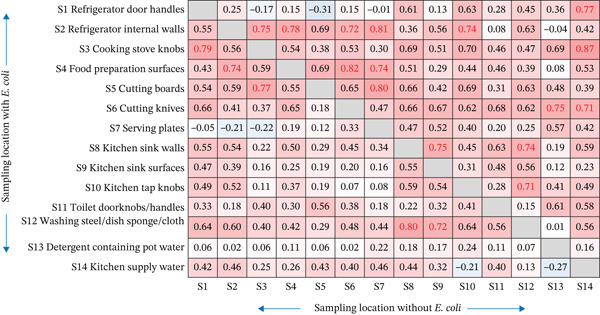
Correlation analysis with and without *E. coli* in household items.

Furthermore, the pattern by which *E. coli* may be transferred among household items was examined. It was assumed that the pattern of *E. coli* at the transmission destination was identical to that of the transmission origin. Thus, only household items with *E. coli* at the origin were included in this analysis. The results demonstrated that *E. coli* can transfer between sampling points with a strong correlation (correlation coefficient > 0.7) (Figure 5).

## 4. Discussion

The household kitchen has traditionally been associated with food preparation, and a typical kitchen comprises a wide range of gadgets and appliances, from small items such as spoons, knives, and cutting boards to large appliances such as microwave ovens, dishwashers, and cookers. Because the house is a possible source of foodborne diseases, surveys have been done to investigate several elements of bacterial contamination in the home. A variety of factors could contribute to contamination of food cooked at home, including food handler failures, poor hygiene, incorrect food storage, and insufficient food preparation circumstances and procedures [18]. Sinclair and Gerba [28] found that the kitchen was more polluted with fecal and coliform bacteria than the bathroom, meaning that the kitchen is the most likely to spread infection in the home. Microbial examinations in family kitchens revealed the presence of a variety of bacterial pollutants, including fecal coliforms, *E. coli*, *Salmonella*, *Staphylococcus* spp., and other enteric bacteria [29–31]. As the residential kitchen is not a designated food production facility, people, food, drinks, pets, insects, and the air all introduce pathogenic and nonpathogenic organisms into the home on a daily basis. Furthermore, people’s food shopping habits play an important role in transferring microbiological pollutants into the home kitchen. In Bangladesh, individuals often buy unwrapped individual horticultural products from wet marketplaces and place them in shopping bags, with green vegetables at the top, allowing water droplets from leafy vegetables to cross‐contaminate all other horticultural products in the bag. The traditional method is to empty the contents of the shopping bags onto the kitchen floor before sorting them for further processing and cooking. The remainder is kept in the refrigerator’s vegetable box, meaning that bringing bacteria from marketplaces into the home and storing them in the refrigerator have the potential to contaminate any product, cooked or uncooked. Washing and disinfecting all purchased vegetables or wrapping them before storing them in the refrigerator should reduce the microbiological risk; nevertheless, this is not a usual practice in Bangladesh. Although washing and cleaning are common in Bangladesh, no food sanitizer has yet been introduced; as a result, bacteria persist in kitchen settings, and householders typically disregard them due to a lack of food safety awareness, understanding, and practice. All of these factors highlight the need to investigate acceptable food preparation processes in home settings, improve food handling standards, and avoid foodborne occurrences at home [10]. To improve home food safety, effective decontamination procedures must be developed, and nonchlorine sanitizing agents appropriate to food and food contact surfaces should be introduced. However, the presence of food ingredients or other debris may reduce the sanitizer’s effectiveness as a decontaminant. Furthermore, the success of a product in eliminating microbes is determined by how the product is administered by the user, as customers do not always use the product as the manufacturer instructs [32]. Nonetheless, it has been recognized that proper cleanliness practices can almost completely eliminate the risk of foodborne pathogen transmission, and consumer food hygiene education and awareness play an important role in preventing foodborne illnesses because they are the final food handlers before consumption. Machado et al. [33] found that proper food handling education was less common in schools, and people lacked food preparation skills. This knowledge is becoming increasingly relevant, underlining the importance of discussing, teaching, and advocating for government regulations that improve home food safety. We must not only introduce new approaches but also reinforce them on a regular basis to ensure that activities are remembered and integrated into the household routine.

## 5. Conclusion

The study results demonstrated that all the common food contact surfaces in the kitchen (e.g., food preparation surfaces, cutting boards, cutting knives, kitchen sink surfaces, kitchen sink walls, and serving plate surfaces), frequently touched surfaces (refrigerator door handles and interior surfaces, kitchen stove knobs, kitchen tap knobs, and toilet doorknobs), and frequently used supplies (kitchen supply water, sponges/dishcloths, and detergent bars) were found to be contaminated with higher TABC, coliform, and *E. coli* levels. The majority of the sampled households (93.3%–100.0%) lacked access to hot water, soap, and sanitizers in the kitchen, which are necessary for thoroughly washing hands, kitchen utensils, and food preparation surfaces. The presence of pets (cats; 6.7% of homes) or pests (100% of homes) in the kitchen was evident, which may increase the likelihood of contamination and the transfer of bacteria from the outside environment to the home. The cleaning detergent bars were found to be contaminated with a variety of microorganisms, including fecal bacteria. Furthermore, *E. coli* was found in kitchen items such as cutting boards, cutting knives, and serving plates, raising the possibility of cross‐contamination and transmission to humans. To avoid bacterial transmission, cleaning detergents or kitchen sanitizers, as well as consumer hygiene education, are necessary.

### 5.1. Limitation of This Study

Because of the population’s cultural sensitivity, the household selection strategy was mainly reliant on convenience sampling (relatives, friends, and families of researchers and students). As a result, the findings cannot be interpreted as typical of all urban households in Dhaka.

## Author Contributions

**Sharmin Zaman:** investigation, methodology, project administration, validation, writing – original draft. **Md. Ashfaq Aziz:** methodology. **Shiori Katta:** visualization, writing – review and editing. **Yuna Takimoto:** funding acquisition, writing – review and editing. **Junichi Sugiyama:** funding acquisition. **Yasushi Kakizawa:** funding acquisition. **Md. Latiful Bari:** conceptualization, funding acquisition, resources, supervision, writing – review and editing.

## Funding

No funding was received for this manuscript.

## Conflicts of Interest

The authors declare no conflicts of interest.

## Data Availability

The data that support the findings of this study are available from the corresponding author upon reasonable request.
